# BCI inhibits MKP3 by targeting the kinase-binding domain and disrupting ERK2 interaction

**DOI:** 10.1016/j.jbc.2025.110570

**Published:** 2025-08-06

**Authors:** Su-Jie Qiu, Ya-Liang Zhang, Wei-Bin Gong, Yu-Han Ding, Jia-Wei Wu, Zhi-Xin Wang, Hong-Wei Yao

**Affiliations:** 1 Institute of Molecular Enzymology, School of Life Sciences, Suzhou Medical College of Soochow University, Suzhou, Jiangsu, P.R. China; 2 MOE Key Laboratory of Geriatric Diseases and Immunology, Suzhou Medical College of Soochow University, Suzhou, Jiangsu, P.R. China; 3 State Key Laboratory of Pharmaceutical Biotechnology, School of Life Sciences, Nanjing University, Nanjing, Jiangsu, P.R. China; 4 Institute of Biophysics, Chinese Academy of Sciences, Beijing, P.R. China

**Keywords:** mitogen-activated protein kinase, MAPK phosphatase 3, dual-specificity phosphatase 6, extracellular signal–regulated kinase, BCI inhibitor

## Abstract

Mitogen-activated protein kinase phosphatase 3 (MKP3), also known as dual-specificity phosphatase 6, is a critical regulator of extracellular signal–regulated kinase (ERK) signaling, and its dysregulation is implicated in diseases, such as cancer. The small-molecule inhibitor BCI ((E)-2-benzylidene-3-(cyclohexylamino)-2,3-dihydro-1H-inden-1-one) has been reported to inhibit MKP3, thereby enhancing ERK signaling and promoting selective cytotoxicity in cancer cells. However, the molecular mechanism underlying BCI-mediated MKP3 inhibition remains unclear. In this research, we characterized the interaction between BCI and MKP3 using NMR titration, microscale thermophoresis, enzymatic assays, and AlphaFold 3 modeling. Our results demonstrate that BCI selectively binds to the kinase-binding domain (KBD) of MKP3, rather than its catalytic domain, thereby disrupting the MKP3–ERK2 interaction and impairing MKP3 activation. Enzymatic assays further reveal that BCI significantly reduces ERK2-mediated MKP3 activity without directly interfering with substrate binding at the active site. AlphaFold 3 structural modeling suggests that BCI binding induces local conformational changes, notably an outward shift of the α4-helix, which exposes a hydrophobic pocket essential for BCI accommodation. Moreover, BCI exhibits differential binding affinities across the MKP family, showing significant interactions with the KBDs of MKPX and MKP5 but markedly weaker or negligible binding to those of MKP1, MKP2, and MKP4. Together, these findings uncover a novel KBD-targeting mechanism of MKP3 inhibition by BCI and highlight the potential of selectively modulating mitogen-activated protein kinase phosphatases through allosteric disruption of kinase–phosphatase interactions. This strategy may offer a new avenue for the design and optimization of targeted phosphatase inhibitors.

Mitogen-activated protein kinases (MAPKs) regulate essential cellular processes, including proliferation, differentiation, apoptosis, and stress responses (1). Dysregulation of MAPK signaling contributes to various human diseases, such as cancer, inflammation, and immune disorders (2, 3, 4, 5). MAPK activity is negatively regulated by mitogen-activated protein kinase phosphatases (MKPs), a subgroup of the dual-specificity phosphatases (DUSPs) that dephosphorylate the Thr-X-Tyr motif within the MAPK activation loop (6, 7). MKPs are classified into three subfamilies based on sequence homology, subcellular localization, and substrate specificity. The first subfamily (MKP1, PAC1, MKP2, and hVH3) primarily localizes to the nucleus and dephosphorylates p38 MAPK, c-Jun N-terminal kinase (JNK), and extracellular signal–regulated kinase (ERK). The second subfamily (MKP3, MKPX, and MKP4) is predominantly cytoplasmic and selectively targets ERKs. The third subfamily (hVH5, MKP5, and MKP7) localizes to both the cytoplasm and nucleus and primarily inactivates JNK and p38 MAPKs.

All MKPs contain an N-terminal kinase-binding domain (KBD) and a C-terminal catalytic domain (CD) (6, 7). In the absence of MAPK substrates, MKPs exhibit varying basal activity levels, which increase significantly upon substrate binding, particularly in the second MKP subfamily (6, 8). For example, ERK2 binding enhances MKP3 and MKPX activity by ∼30-fold and MKP4 activity by ∼4-fold (9, 10). Even in subfamilies with higher basal activity, substrate binding enhances activity ∼2-fold (11, 12, 13). MAPKs activate MKPs through interactions with the KBD and/or CD. ERK and p38 MAPKs primarily bind the kinase interaction motif within the KBD, whereas JNK interacts with the highly conserved Phe-X-Phe motif in the CD (14).

Although the full-length structures of MKPs (MKP^FL^) remain unresolved, structural studies have characterized MKP3^KBD^, MKP5^KBD^, and MKP7^KBD^ using NMR spectroscopy and X-ray crystallography (15, 16, 17). Despite sharing ∼30% sequence identity, MKP3^KBD^’s NMR structure differs significantly from the crystal structures of MKP5^KBD^ and MKP7^KBD^. MKP CDs are highly conserved (37–89% sequence identity) and exist in low- and high-activity conformations, distinguished by the orientation of key active-site residues (18). For instance, the low-activity MKP3^CD^ structure shows the Arg in the P-loop and Asp in the D-loop positioned away from the active site (19), whereas the high-activity MKPX^CD^ and MKP5^CD^ structures show these residues oriented toward the active site (17, 20, 21). Notably, AlphaFold 2 (AF2) predicts a high-activity conformation for MKP3 (22, 23), differing from its crystal structure (Fig. 1, *A*–*C*). Molecular dynamics simulations suggest that the transition between low- and high-activity states involves structural rearrangements, including the conversion of the extra β3′-strand in the low-activity conformation into a random coil (18).

**Figure 1:**
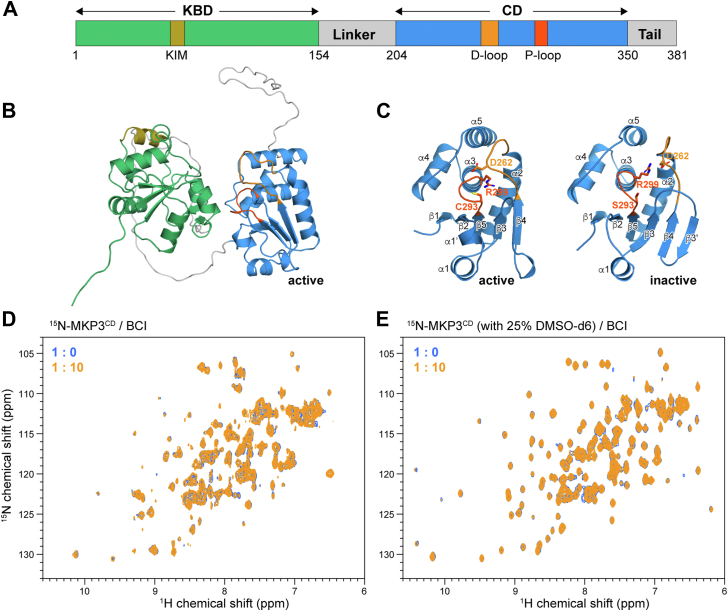
**NMR titrations of the MKP3 catalytic domain (CD) with BCI**. *A*, schematic representation of the domain structure of MAPK phosphatase MKP3. *B*, the AF2-predicted structure of full-length human MKP3 (MKP3^FL^). The CD (*blue*) is shown in its active state with the D-loop (*light orange*) positioned close to the P-loop (*dark orange*). *C*, the X-ray crystal structure of the MKP3^CD^ in its inactive state (Protein Data Bank ID: 1HZM) (*right*). Compared with the active state (*left*), the D-loop is positioned farther from the P-loop, and an additional β3′-strand forms from the β3–β4 loop. *D* and *E*, NMR titrations of MKP3^CD^ with BCI in the absence (inactive state) and presence (active state) of deuterated DMSO. Spectra recorded without and with BCI are shown in *blue* and *orange*, respectively. The MKP3 inhibitor BCI does not perturb the conformation of MKP3^CD^ in either its inactive or active states. AF2, AlphaFold 2; BCI, (*E*)-2-benzylidene-3-(cyclohexylamino)-2,3-dihydro-1*H*-inden-1-one; DMSO, dimethyl sulfoxide; MAPK, mitogen-activated protein kinase; MKP3, mitogen-activated protein kinase phosphatase 3.

Given their role in MAPK regulation and disease pathogenesis, MKPs are promising therapeutic targets (24, 25). However, their highly conserved CDs have made the development of selective inhibitors challenging, leading to their classification as “undruggable” (26, 27, 28). Recent advances have enabled the discovery of allosteric MKP inhibitors, including BCI [(*E*)-2-benzylidene-3-(cyclohexylamino)-2,3-dihydro-1*H*-inden-1-one] and its analogs, which target MKP3, and compound 1, which selectively inhibits MKP5 (7, 29, 30, 31). The crystal structure of MKP5^CD^ bound to compound 1 reveals an allosteric binding site distinct from the active site, providing a basis for selective inhibition (31). BCI, identified through a zebrafish chemical screen, selectively inhibits MKP3, leading to ERK1/2 hyperactivation and inducing cancer cell death (29, 32, 33). In addition, BCI-215, an analog of BCI, has demonstrated selective cytotoxicity against B-cell acute lymphoblastic leukemia (B-ALL) and breast cancer cells (30, 34, 35). Despite these findings, the precise molecular mechanism of BCI-mediated MKP3 inhibition remains unclear because of the lack of structural biology evidence.

In this study, we characterized the interaction between MKP3 and BCI using NMR titration, microscale thermophoresis (MST), enzymatic assays, and AlphaFold 3 (AF3) modeling. Our results show that BCI selectively binds to the KBD of MKP3 rather than the CD. The AF3-predicted structure of the MKP3^KBD^–BCI complex aligns well with the NMR-identified binding residues and suggests that BCI binding induces a shift in the α4-helix, exposing a hydrophobic pocket that stabilizes the interaction. We further evaluated BCI’s selectivity across multiple MKP KBDs and found that it binds significantly to MKPX and MKP5 but exhibits weak or no interaction with MKP1, MKP2, and MKP4. Sequence and structural analyses suggest that variations within the α4-helix and its surrounding regions likely underlie this selectivity. Collectively, these findings highlight the KBD as a promising and structurally diverse alternative target for MKP inhibition, in contrast to the highly conserved CD.

## Results

### BCI does not bind to the CD of MKP3

Docking simulations by Tsang *et al*. suggested that BCI inhibits MKP3 by binding to an allosteric site within the CD (29, 30). However, this predicted binding region had not been experimentally validated. To address this, we conducted NMR titration experiments. Unexpectedly, the addition of excess BCI did not perturb the NMR spectrum of MKP3^CD^ (Fig. 1*D*), indicating no detectable interaction. Given that free MKP3^CD^ predominantly exists in a low-activity state (18, 19), we considered whether BCI might preferentially interact with the high-activity conformation. To assess this, we induced a conformational shift toward the high-activity state by adding 25% dimethyl sulfoxide (DMSO), which is known to promote the transition between inactive and active MKP3 states (36). As expected, in the presence of DMSO, the MKP3^CD^ spectrum exhibited narrower linewidths with improved dispersion (Fig. 1*E*), consistent with a more homogeneous and active conformation. However, even under these conditions, BCI still failed to perturb the spectrum of MKP3^CD^ (Fig. 1*E*), suggesting that it does not interact with MKP3^CD^ in either the low- or high-activity states.

### BCI selectively binds to the KBD of MKP3

**Figure 2:**
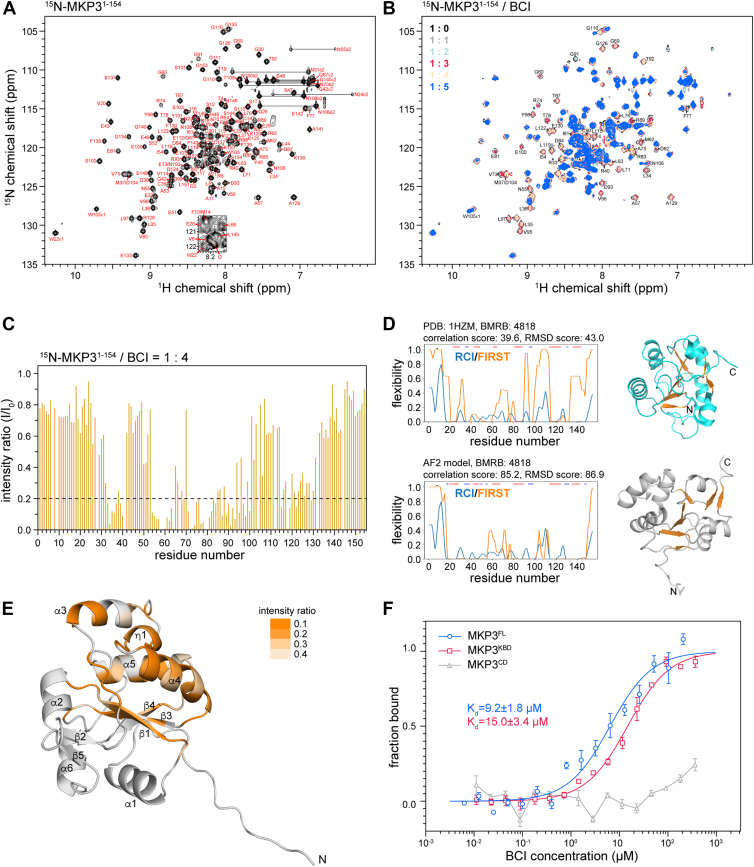
**Interaction of the kinase-binding domain (KBD) of MKP3 with BCI**. *A*, 2D ^1^H–^15^N HSQC spectrum of the MKP3 KBD (MKP3^1–154^, residues 1–154). Residue assignments are labeled using one-letter codes. *B*, NMR titration of MKP3^1–154^ with BCI. A large number of residue peaks were significantly broadened by BCI. *C*, plot of intensity ratios *versus* residue number. The spectrum of a 1:4 M ratio of ^15^N-MKP3^1–154^ to BCI was analyzed. *D*, ANSURR analysis of the NMR structure and AF2 model of the MKP3 KBD. *Blue lines* represent rigidity computed from the random coil index (RCI) based on the backbone chemical shifts of MKP3^1–154^ (BMRB: 4818), whereas *orange lines* depict rigidity derived from the structure using rigidity theory (FIRST). The AF2 model (*gray*) demonstrates higher correlation and a better RMSD score compared with the NMR structure (*cyan*), indicating greater structural accuracy. Although shown separately, the two structures were aligned, and β-strands are colored *orange* in both to facilitate visual comparison. *E*, mapping of perturbed residues onto the AF2 structure of MKP3^1–154^ based on intensity ratios from (*C*). *F*, affinity measurements by microscale thermophoresis (MST). Data represent the mean of three independent experiments, with error bars indicating the SD. *K*_*d*_ values were obtained by fitting to a one-site binding model, and the SD was calculated from the *K*_*d*_ values derived from each independent experiment. Both the KBD (MKP3^KBD^) (*red*) and MKP3^FL^ (*blue*) exhibit similar binding affinities to BCI. In contrast, no detectable binding was observed between MKP3^CD^ (*gray*) and BCI. AF2, AlphaFold2; ANSURR, Accuracy of NMR Structures using Random Coil Index and Rigidity; BCI, (*E*)-2-benzylidene-3-(cyclohexylamino)-2,3-dihydro-1*H*-inden-1-one; BMRB, Biological Magnetic Resonance Bank; FIRST, Floppy Inclusions and Rigid Substructure Topography; HSQC, heteronuclear single quantum coherence; MKP3, mitogen-activated protein kinase phosphatase 3.

Previous phosphatase assays showed that while BCI does not affect MKP3’s basal activity toward 3-*O*-methylfluorescein phosphate (OMFP), it inhibits ERK2-mediated MKP3 activity (29, 30). This inhibition was initially interpreted as evidence supporting BCI as an allosteric inhibitor acting on the CD. However, since ERK2 primarily activates MKP3 through binding to the KBD of MKP3, an alternative possibility is that BCI disrupts this interaction. Based on this, we hypothesized that BCI may directly target MKP3’s KBD. To test this, we performed an NMR titration of ^15^N-labeled MKP3^KBD^ with BCI. Backbone chemical shifts of MKP3^KBD^ were assigned using 3D CBCA(CO)NH, HNCACB, HNCO, and HN(CA)CO spectra, with assigned residues indicated in the 2D ^1^H–^15^N heteronuclear single quantum coherence (HSQC) spectrum (Fig. 2*A*). Notably, BCI caused significant peak broadening in the MKP3^KBD^ spectra (Fig. 2*B*), indicating a direct interaction, in contrast to our observations with MKP3^CD^, where no interaction was detected. By analyzing peak intensity changes in the spectra of MKP3^KBD^ in the absence and presence of BCI at a 1:4 M ratio, the most affected regions were identified as residues 33 to 40, 54 to 63, 71 to 84, 87 to 100, and 115 to 129 (Fig. 2*C*).

To structurally map the binding site, we first evaluated the accuracy of the available NMR structure of MKP3^KBD^ using the ANSURR (Accuracy of NMR Structures using Random Coil Index and Rigidity) method (37, 38), as it deviates significantly from the AF2-predicted model. Recent studies using ANSURR suggest that many solution NMR structures are less accurate than AF2 predictions (39). By ANSURR, local backbone rigidity (random coil index) of MKP3^KBD^ was calculated from its deposited chemical shifts (Biological Magnetic Resonance Bank: 4818) and compared with the rigidities of the AF2 model and NMR structure, respectively, as computed using FIRST (Floppy Inclusions and Rigid Substructure Topography) program (Fig. 2*D*). Surprisingly, the AF2 model showed both a stronger correlation score with local rigidity and a higher overall rigidity RMSD score than the NMR structure. As only a single conformer was deposited in the Protein Data Bank, that structure was used for the ANSURR analysis. Based on its superior rigidity agreement, we selected the AF2 model for subsequent analysis of the MKP3–BCI interaction.

Mapping the perturbed residues onto the AF2 model of MKP3^KBD^ revealed a binding pocket formed by the α3, α4, and α5 helices, the η1-helix (a short 3_10_-helix between α3 and α4), the β1, β3, and β4 strands, and the β1–α2 and β2–α3 loops (Fig. 2*E*). However, this pocket appears to remain closed because of obstruction by the α4-helix. To assess BCI binding affinity, we performed MST (Fig. 2*F*). The dissociation constants (*K*_*d*_) for FL MKP3 (MKP3^FL^) and MKP3^KBD^ with BCI were 9.2 ± 1.8 μM and 15.0 ± 3.4 μM, respectively, which are comparable, confirming that the interaction primarily occurs through MKP3^KBD^. In contrast, no binding was detected between BCI and MKP3^CD^.

The significant line broadening observed in the NMR spectra upon BCI titration, in the absence of clear chemical shift perturbations, is most consistent with intermediate exchange dynamics on the μs–ms timescale. Such a behavior is commonly observed in systems where ligand binding induces local conformational changes or when the ligand interacts with moderate affinity—typically in the low to mid micromolar range, consistent with our MST-derived *K*_*d*_ values. Importantly, the signal attenuation was localized rather than global, affecting specific regions of MKP3^KBD^—particularly residues 33 to 40, 54 to 63, 71 to 84, 87 to 100, and 115 to 129. This pattern suggests that the broadening arises from local conformational exchange at or near the BCI binding interface, rather than from nonspecific aggregation or global structural perturbation.

Together, these findings demonstrate that BCI selectively binds to the KBD of MKP3, likely blocking ERK2-mediated activation rather than directly inhibiting the CD.

### The primary binding sites of BCI and ERK2 on MKP3^KBD^ are distinct yet spatially proximal

**Figure 3:**
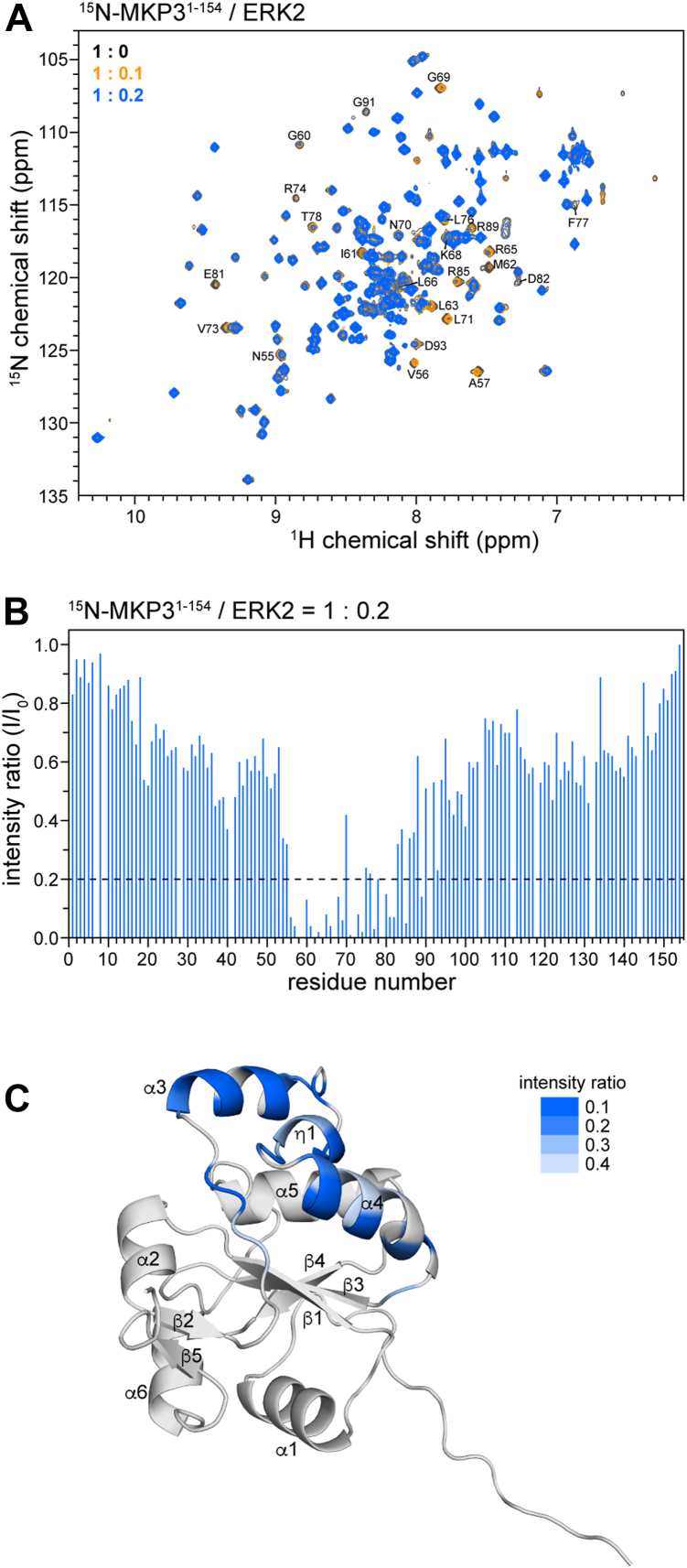
**NMR titration of MKP3^KBD^ with ERK2**. *A*, 2D ^1^H–^15^N HSQC spectra of MKP3^1–154^ in the absence (*black*) and presence (*orange* and *blue*) of ERK2. Significant peak broadening was observed for many residues upon the addition of ERK2. *B*, plot of intensity ratios *versus* residue number. The spectrum corresponding to a 1:0.2 M ratio of ^15^N-MKP3^1–154^ to ERK2 was analyzed. *C*, the binding sites were mapped onto the AF2 model of MKP3^1–154^ based on the intensity ratios from (*B*). AF2, AlphaFold 2; ERK2, extracellular signal–regulated kinase 2; HSQC, heteronuclear single quantum coherence; KBD, kinase-binding domain; MKP3, mitogen-activated protein kinase phosphatase 3.

To compare the binding sites of ERK2 and BCI on MKP3^KBD^, we performed NMR titration of ^15^N-labeled MKP3^KBD^ with ERK2. As expected, several MKP3^KBD^ peaks were significantly broadened upon ERK2 binding (Fig. 3*A*), likely because of conformational exchange occurring on the μs–ms timescale. A plot of peak intensity ratios *versus* residue number was generated from spectra of free MKP3^KBD^ and MKP3^KBD^ in a 1:0.2 M ratio with ERK2 (Fig. 3*B*). The broadening signals originated from residues 56 to 89, located in the β2–α3 loop, α3-helix, η1-helix, and α4-helix (Fig. 3*C*). Among these, the α3-helix, which forms part of the kinase interaction motif (residues 59–74) (40), was identified as the primary ERK2-binding region based on the extent and pattern of peak intensity reduction. In contrast, perturbations observed in the β2–α3 loop, η1-helix, and α4-helix, likely resulted from conformational changes induced by ERK2 binding.

In comparison, BCI binding predominantly perturbed the β2–α3 loop, η1-helix, α4-helix, and the β1-strand, suggesting that these regions form the primary BCI-binding interface. Perturbations observed in other areas, including the α3-helix, are likely secondary effects resulting from BCI-induced conformational changes. Mapping the perturbed residues onto the MKP3^KBD^ structure revealed that the ERK2 and BCI binding regions are spatially adjacent and partially overlapping—particularly within residues 54 to 63 and 71 to 84. However, residues 65 to 69, including the critical Arg65, were minimally affected by BCI (Arg64 overlapped with S144 and was excluded from analysis), yet showed strong perturbation upon ERK2 binding. This distinction suggests that although the two binding regions are spatially close and share some overlap, their primary interaction surfaces are distinct.

To further investigate conformational dynamics in these regions, we conducted ^15^N spin relaxation experiments, including measurements of T_1_, T_2_, and ^1^H–^15^N steady-state NOE (Fig. S1). The average NOE value for the α3-helix was approximately 0.71, lower than those observed for other secondary structural elements (ranging from 0.77 to 0.84), indicating reduced rigidity. This finding was further supported by relatively elevated R_1_ values, consistent with enhanced fast-timescale (ps–ns) internal motions. Notably, the η1-helix (residues 74–76) and adjacent residue 77 exhibit elevated R_2_ values while maintaining R_1_ and NOE values similar to those of more rigid regions—a pattern indicative of conformational exchange on the μs–ms timescale. Similarly, the C-terminal segment of the α4-helix (residues 89–91) displayed increased R_2_ values relative to the rest of the helix, suggesting contributions from μs–ms exchange.

Together, these data indicate that the α3-helix, η1-helix, and the flanking regions of the α4-helix undergo localized, site-specific conformational dynamics that likely facilitate selective ligand or substrate binding. ERK2 and BCI appear to bind at distinct but spatially adjacent regions on MKP3^KBD^, potentially leading to steric interference when both are present.

### BCI disrupts the ERK2–MKP3^KBD^ interaction

**Figure 4:**
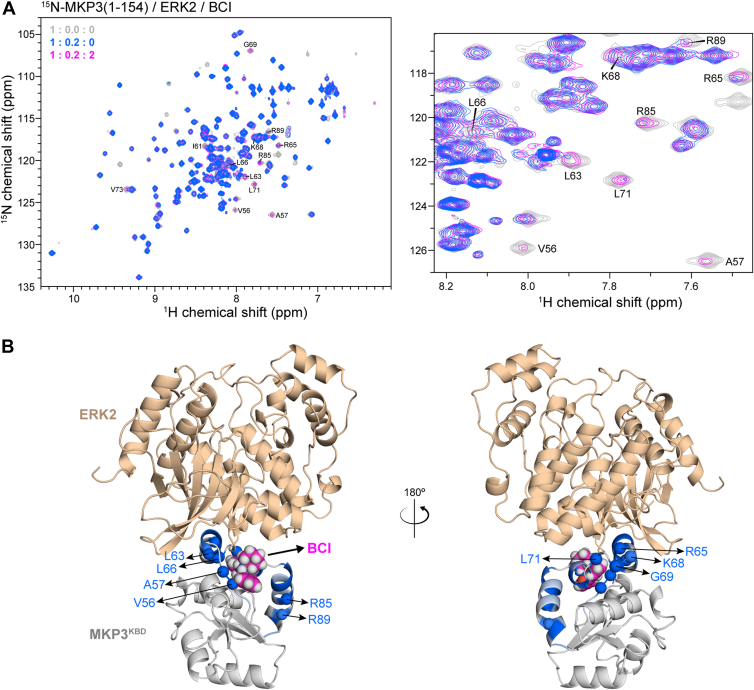
**NMR competitive binding titration of ERK2 and BCI to MKP3^KBD^**. *A*, 2D ^1^H–^15^N HSQC spectra of ^15^N-MKP3^1–154^ in the presence of ERK2 without (*blue*) and with (*magenta*) the addition of BCI. The spectrum of free-state ^15^N-MKP3^1–154^ is shown as a reference (*gray*). The addition of excess BCI restores most of the peaks broadened by ERK2. For clarity, a portion of the spectra is zoomed in. *B*, structural overlay of the AF2-predicted MKP3^KBD^–ERK2 complex and the docking model of the MKP3^KBD^–BCI complex. Residues perturbed by ERK2 are highlighted in *blue* on the MKP3^KBD^ structure, whereas residues restored by BCI are depicted as *spheres* representing the backbone alpha-carbon. To simplify visualization, only one MKP3^KBD^ structure (*gray*) is displayed. AF2, AlphaFold 2; BCI, (*E*)-2-benzylidene-3-(cyclohexylamino)-2,3-dihydro-1*H*-inden-1-one; ERK2, extracellular signal–regulated kinase 2; HSQC, heteronuclear single quantum coherence; KBD, kinase-binding domain; MKP3, mitogen-activated protein kinase phosphatase 3.

To investigate whether BCI interferes with ERK2 binding to MKP3^KBD^, we performed NMR competitive binding titrations. As expected, the addition of excess BCI to the MKP3^KBD^ and ERK2 complex resulted in the reversion of specific MKP3^KBD^ peaks to their ERK2-unbound state (Fig. 4*A*). Notably, residues V56, A57, L63, R65, L66, K68, G69, L71, R85, and R89 exhibited this reversion. Among them, R65, L66, K68, and G69, located in the C-terminal portion of the α3-helix, were significantly broadened by ERK2 but remained unaffected by BCI, suggesting that BCI primarily disrupts ERK2 binding at these key residues.

In contrast, residues V56, A57, L63, L71, R85, and R89, located in the β2–α3 loop, the N-terminal portion of the α3-helix, the α3-η1 loop, and the α4-helix, were perturbed by both ERK2 and BCI. The reversion of these peaks upon BCI addition primarily resulted from the dissociation of ERK2, along with the low affinity of BCI for MKP3^KBD^ and the relatively low BCI concentration used in the titration, which may have been insufficient to cause significant spectral perturbations. Structural analysis, integrating the AF2 model of the MKP3^KBD^–ERK2 complex with a docking model of the MKP3^KBD^–BCI complex, revealed that BCI binds to a site distinct from ERK2 (Fig. 4*B*). This spatial separation suggests that BCI may sterically hinder the ERK2–MKP3^KBD^ interaction. Consequently, BCI likely inhibits ERK2-induced MKP3 phosphatase activity by disrupting its binding to MKP3’s KBD.

To further validate the specificity of BCI binding, we generated a series of MKP3 mutants targeting residues within the α4-helix—E81G, R83G, R85G, T87G, and R89A—since this region is directly involved in BCI binding but not ERK2 interaction. Enzymatic assays using *p*-nitrophenyl phosphate (pNPP) in the presence of ERK2 showed that all mutants retained ERK2-dependent activation (Fig. S2), indicating preserved ERK2 binding. Upon BCI addition, mutants E81G, R83G, and R89A displayed higher enzymatic activity than WT MKP3 (MKP3^WT^), though not fully restored to the BCI-free level. In contrast, R85G and T87G showed BCI inhibition comparable to MKP3^WT^ (Fig. S2). These findings suggest that E81, R83, and R89 are important for BCI-mediated inhibition. MST binding assays further supported these conclusions: MKP3^E81G^, MKP3^R83G^, and MKP3^R89A^ exhibited significantly reduced affinity for BCI compared with MKP3^WT^, whereas MKP3^R85G^ and MKP3^T87G^ bound BCI with affinities similar to the WT (Fig. S3).

Together, these results indicate that BCI and ERK2 bind to distinct primary regions of MKP3^KBD^. Although none of the mutations completely abolished BCI binding—likely because of predominant hydrophobic interactions with a surrounding residue cluster—they nonetheless provide additional evidence for the presence of separate primary binding sites for BCI and ERK2 on MKP3.

### BCI inhibits MKP3 phosphatase activity by disrupting the ERK2–MKP3^KBD^ interaction

To evaluate the impact of BCI on MKP3 phosphatase activity, we conducted enzymatic assays using both the small substrate analog pNPP and the native substrate phosphorylated ERK2 (pERK2), phosphorylated at residues T183 and Y185. pNPP binds directly to MKP3’s catalytic pocket for dephosphorylation but does not interact with the KBD. In contrast, ERK2 can bind to MKP3 at either the KBD or the CD, with a stronger interaction at the KBD, significantly enhancing MKP3’s dephosphorylation activity (15, 18, 41).

**Figure 5:**
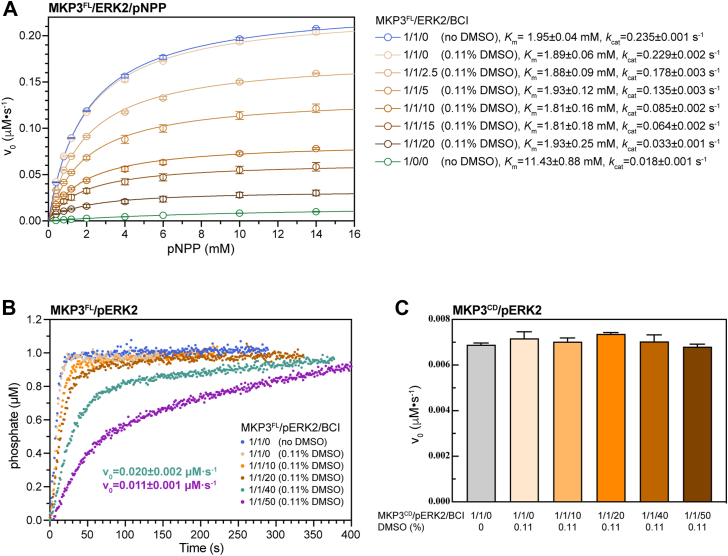
**Inhibition of MKP3 activity by BCI**. *A*, inhibition of MKP3^FL^ activity by BCI toward the pNPP substrate in the presence of ERK2. Initial velocities (*v*_0_) are shown as the mean of three independent experiments, with error bars representing the SD. Errors for *k*_cat_ and *K*_*m*_ indicate the SEs from nonlinear regression fitting. BCI significantly inhibited the ERK2-induced MKP3 activity toward pNPP. *B*, inhibition of MKP3^FL^ activity by BCI toward the phosphorylated ERK2 (pERK2) substrate. BCI effectively slowed the dephosphorylation of pERK2. *C*, initial velocities of MKP3^CD^ in the dephosphorylation of pERK2 in the absence and presence of BCI (mean ± SD, n = 3). BCI had no effect on the MKP3^CD^ activity toward pERK2. To maintain consistency in the reactions with varying molar ratios of MKP3 to BCI, stock solutions of BCI were prepared to ensure the same DMSO concentration (0.11%) across all reactions. Kinetic parameters were derived by fitting the data to the Michaelis–Menten equation (*v*_0_ = *k*_cat_[E][S]/(*K*_*m*_ + [S])). BCI, (*E*)-2-benzylidene-3-(cyclohexylamino)-2,3-dihydro-1*H*-inden-1-one; DMSO, dimethyl sulfoxide; ERK2, extracellular signal–regulated kinase 2; MKP3, mitogen-activated protein kinase phosphatase 3; MKP3^FL^, full-length MKP3; pERK2, phosphorylated ERK2; pNPP, *p*-nitrophenyl phosphate.

Enzymatic assays revealed that when ERK2 was present at a 1:1 M ratio with MKP3^FL^, MKP3’s dephosphorylation activity toward 14 mM pNPP increased to 21 times its basal level (*v*_0_) (Fig. 5*A*). However, BCI significantly reduced ERK2-induced MKP3 activity, with a molar concentration 20 times that of ERK2 lowering MKP3 activity to one-seventh of its initial level. Notably, BCI did not significantly alter MKP3’s Michaelis constant (*K*_*m*_) for pNPP, indicating that BCI does not affect pNPP binding to the active site (Fig. 5*A*). These findings suggest that BCI inhibits MKP3 activity by disrupting its interaction with ERK2 rather than directly blocking substrate binding at the catalytic center. Consistent with this, BCI significantly slowed pERK2 dephosphorylation by MKP3^FL^, with a concentration 50 times that of pERK2 extending the dephosphorylation time by more than 20-fold (Fig. 5*B*). In contrast, BCI had no effect on MKP3^CD^ activity toward pERK2 (Fig. 5*C*), further supporting the conclusion that BCI interferes with ERK2 binding to MKP3’s KBD rather than affecting the CD. In conclusion, BCI inhibits ERK2-induced MKP3 phosphatase activity by disrupting the interaction between ERK2 and MKP3’s KBD.

### BCI exhibits differential affinities for MKP family members through their KBDs

Previous studies have reported that BCI inhibits MKP1 activity by binding to its CD (29, 30). To determine whether BCI interacts with other MKP family members, we examined its binding to the KBDs of MKPX, MKP4, MKP1, MKP2, and MKP5 (MKPX^KBD^, MKP4^KBD^, MKP1^KBD^, MKP2^KBD^, and MKP5^KBD^) as well as the CD of MKP5 (MKP5^CD^).

**Figure 6:**
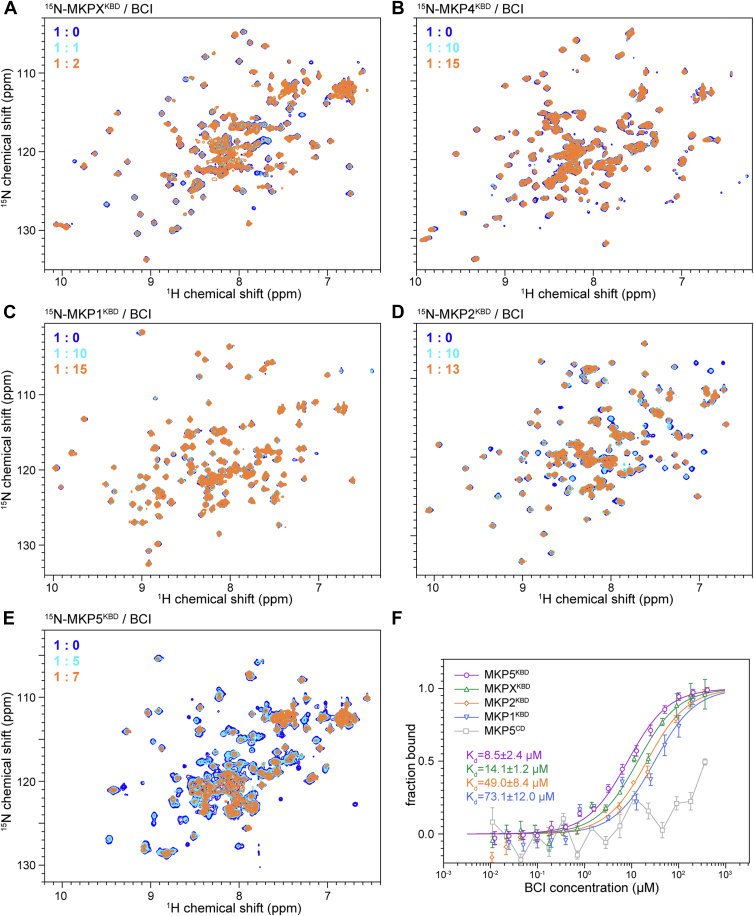
**Interactions between various MKP phosphatases and BCI**. *A*–*E*, 2D ^1^H–^15^N HSQC spectra of the kinase-binding domains (KBDs) of various MKP phosphatases in the absence (*blue*) and presence of BCI (*cyan* and *orange*). Upon the addition of BCI, significant peak broadening was observed for MKPX^KBD^, MKP2^KBD^, and MKP5^KBD^, indicating strong interactions. In contrast, the spectra of MKP4^KBD^ and MKP1^KBD^ were almost unaffected even at high BCI concentrations. *F*, affinity measurements by MST. Data represent the mean of three independent experiments, with error bars indicating the SD. *K*_*d*_ values were obtained by fitting to a one-site binding model, and the SD was calculated from the *K*_*d*_ values derived from each independent experiment. The binding affinities of MKP5^KBD^ (*purple*), MKPX^KBD^ (*green*), MKP2^KBD^ (*orange*), and MKP1^KBD^ (*blue*) to BCI were determined ranging from ∼8.5 μM to 73.1 μM. No interaction was detected between MKP5^CD^ (*gray*) and BCI. The binding affinity of MKP4^KBD^ to BCI could not be measured because of precipitation observed during MST experiments. BCI, (*E*)-2-benzylidene-3-(cyclohexylamino)-2,3-dihydro-1*H*-inden-1-one; CD, catalytic domain; HSQC, heteronuclear single quantum coherence; MKP, mitogen-activated protein kinase phosphatase; MST, microscale thermophoresis.

NMR titration and MST affinity measurements revealed that BCI interacts notably with MKPX^KBD^ and MKP5^KBD^ (Fig. 6, *A*–*F* and Fig. S4). Significant peak broadening was observed in the NMR spectra at molar ratios of 1:2 and 1:5 for MKPX^KBD^ and MKP5^KBD^, respectively, with affinity constants of approximately 14.1 μM and 8.5 μM, similar to the MKP3^KBD^–BCI interaction (Fig. 6, *A* and *E*, *F*). In contrast, no interaction was detected between BCI and MKP5^CD^. BCI exhibited much weaker interactions with MKP4^KBD^, MKP1^KBD^, and MKP2^KBD^ (Fig. 6, *B*–*D*, *F*). No significant NMR peak perturbation was observed even at a molar ratio of 1:15 for MKP4^KBD^ or MKP1^KBD^. Peak broadening was detected for MKP2^KBD^ only when its molar ratio to BCI reached 1:13, with affinity constants of approximately 49.0 μM and 73.1 μM for MKP2^KBD^ and MKP1^KBD^, respectively. The affinity of MKP4^KBD^ for BCI could not be determined because of visible protein precipitation during MST measurements.

These findings suggest that BCI interacts with multiple MKP family members through their KBDs, exhibiting distinct affinities. Specifically, BCI binds significantly to MKP3^KBD^, MKPX^KBD^, and MKP5^KBD^, whereas its interactions with MKP1^KBD^, MKP2^KBD^, and MKP4^KBD^ are much weaker, highlighting differential specificity and affinity across the MKP family.

### Structural insights into BCI binding to MKP3^KBD^ and selectivity across MKP phosphatases

To elucidate the structural basis of BCI binding to MKP3^KBD^, we attempted to crystallize the MKP3^KBD^–BCI complex along with other MKP^KBD^–BCI complexes. However, crystallization was unsuccessful. As an alternative, we performed docking simulations using fixed MKP^KBD^ structures (Fig. 4*B*). The results suggested that BCI binds to a shallow surface pocket on MKP3^KBD^, primarily involving the α2-helix, β2–α3 loop, and α3–α4 loop. This predicted binding site partially overlaps with the region identified by NMR titration, though most residues differ, suggesting that BCI may initially bind at the entrance of a pocket that undergoes conformational changes upon complex formation.

**Figure 7:**
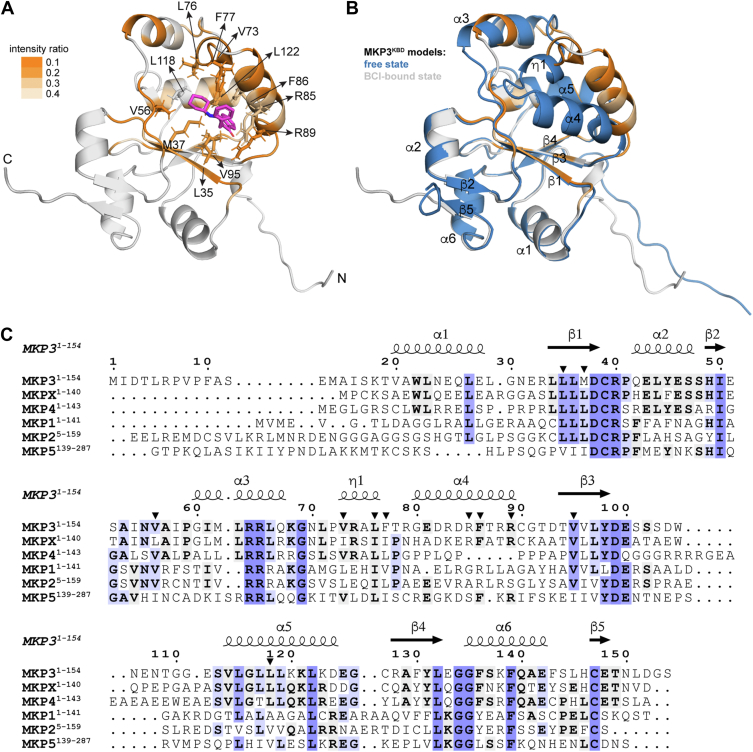
**AF3 model of the MKP3^KBD^–BCI complex**. *A*, AF3 model of the MKP3^KBD^–BCI complex. Residues perturbed by BCI were mapped onto the structure model based on peak intensity ratios (I/I_0_) before and after the addition of BCI in NMR titration. BCI is depicted as *sticks* with carbon, nitrogen, and oxygen atoms colored *magenta*, *blue*, and *red*, respectively. The residues contacting BCI were labeled, and their side chains were displayed. *B*, superimposition of MKP3^KBD^ structure models in the free and BCI-bound states. BCI binding induces significant conformational changes in the α4-helix and η1-helix. *C*, sequence alignment of MKP3^KBD^, MKPX^KBD^, MKP4^KBD^, MKP1^KBD^, MKP2^KBD^, and MKP5^KBD^. The secondary structure elements and residue numbers of MKP3^KBD^ are shown at the *top*. The symbols α, β, and η represent α-helix, β-strand, and 3_10_-helix, respectively. The *solid triangles* indicate MKP3^KBD^ residues involved in BCI interaction in the AF3 model (*A*). AF3, AlphaFold 3; BCI, (*E*)-2-benzylidene-3-(cyclohexylamino)-2,3-dihydro-1*H*-inden-1-one; KBD, kinase-binding domain; MKP, mitogen-activated protein kinase phosphatase.

To further investigate this conformational change, we employed AF3 modeling, which predicts protein–ligand complex structures while accounting for ligand-induced structural adjustments (42). Remarkably, the AF3 model of the MKP3^KBD^–BCI complex revealed a binding site that closely aligns with the one identified by NMR titration (Fig. 7*A*). In this model, BCI occupies a hydrophobic pocket formed by the β2–α3 loop, α3–α4 loop, α4-helix, β1-strand, β3-strand, and α5-helix, involving residues L35, M37, V56, V73, L76, F77, R85, F86, R89, V95, L118, and L122. Upon BCI binding, MKP3^KBD^ undergoes a substantial conformational change, primarily within the region spanning V56 to T92 (Fig. 7*B*). This includes structural rearrangements in the β2–α3 loop, α3-helix, α3–α4 loop, and α4-helix, with the most pronounced changes occurring in the α3–α4 loop and α4-helix, facilitating the opening of the hydrophobic pocket and stabilizing BCI binding.

AF3 models of other MKP phosphatases revealed both similarities and differences in BCI binding sites (Fig. S5). While MKP3 and MKP5 share a broadly similar BCI-binding pocket, MKPX, MKP1, and MKP2 exhibit distinct binding regions. Despite structural similarities among MKP KBDs, their sequence identities vary, particularly in regions, such as the α1-helix, α4-helix, and β3–α5 loop, which may contribute to their differential binding specificity for BCI (Fig. 7*C*). Compared with MKP1^KBD^, MKP2^KBD^, and MKP5^KBD^—which share approximately 25% sequence identity with MKP3^KBD^—MKPX^KBD^ and MKP4^KBD^ exhibit much higher sequence identity with MKP3^KBD^, at 59% and 44%, respectively, as they belong to the same subfamily. However, MKP4^KBD^ is enriched in proline residues between the α3-helix and β3-strand, preventing the formation of the α4-helix observed in other KBDs. This structural difference likely accounts for the significantly weaker or undetectable interaction between MKP4^KBD^ and BCI.

These findings suggest that BCI binding induces a conformational change in MKP3^KBD^, stabilizing its interaction within a hydrophobic pocket. The variation in predicted BCI binding sites and observed affinities across MKP family members underscores the structural and sequence-specific determinants of BCI’s selectivity. Although AF3 predicts BCI binding sites on multiple MKPs, experimental validation remains lacking for members other than MKP3. This limits a deeper understanding of BCI’s selective binding, including the specific residues involved and their contributions to binding specificity and affinity. Addressing these questions will be a key focus of future work.

## Discussion

MKPs have emerged as potential therapeutic targets because of their upregulation in various human diseases (7, 43, 44, 45). Among the inhibitors developed, BCI holds the greatest promise in targeting MKP3 (7). By inhibiting MKP3 activity, BCI induces ERK1/2 hyperactivation, promoting cell death in B-ALL and gastric cancer while overcoming drug resistance (32, 33). In addition, its analog, BCI-215, synergistically enhances B-ALL cell death when combined with ruxolitinib (34). However, the precise molecular interaction between MKP3 and BCI remains unclear because of the lack of structural data.

In the original study (29), BCI was identified through a zebrafish screen as a specific inhibitor of DUSP6 (MKP3), but not DUSP5 (hVH3), although both enzymes dephosphorylate pERK. Considering that MKP3 activity is stimulated by substrate binding, whereas hVH3 is constitutively active, it was suggested that BCI might suppress MKP3 activation associated with substrate interaction. Subsequent *in vitro* phosphatase assays showed that BCI could inhibit MKP3 activity, and the effect was similar to that of sodium orthovanadate, a general tyrosine phosphatase inhibitor known to target the catalytic center. This likely prompted the use of the MKP3 CD structure—rather than the KBD—for docking simulations. The docking model positioned BCI in a pocket adjacent to—but not overlapping with—the catalytic site, and the enzymatic assays were interpreted to support this model. For example, the basal phosphatase activity of MKP3 toward OMFP was inhibited by NSC95397 (a catalytic site inhibitor), but not by BCI, suggesting a noncompetitive mechanism. In contrast, BCI suppressed ERK2-stimulated MKP3 activity toward OMFP, leading to the proposal that BCI functions as an allosteric inhibitor that prevents substrate-induced catalytic enhancement. However, an alternative interpretation of these enzymatic data is that BCI may specifically interfere with the interaction between MKP3^KBD^ and ERK2. In the absence of a high-resolution MKP3^CD^–BCI complex structure, the proposed allosteric mechanism involving the CD remains plausible but inconclusive. To address this uncertainty, we investigated the interaction between MKP3 and BCI using NMR titrations, MST affinity measurements, enzymatic assays, and AF3 modeling. Our results demonstrate that BCI primarily binds to the KBD of MKP3 rather than its CD. The primary binding site for BCI on the KBD differs from that of ERK2, yet they are spatially proximal. Furthermore, BCI binding disrupts the ERK2–MKP3^KBD^ interaction, thereby inhibiting ERK2-mediated MKP3 activity. AF3 modeling suggests that BCI binding induces an outward shift of the α4-helix in MKP3^KBD^, creating a hydrophobic pocket that tightly accommodates BCI. In addition, BCI exhibits differential affinities across the KBDs of the MKP family, underscoring its selectivity, which likely arises from sequence or conformational differences in the α4-helix.

The α4-helix plays a central role in BCI’s selective binding to MKP3^KBD^, exhibiting both conformational flexibility and structural specificity. Although the AF2 model does not predict direct interactions between α4-helix residues and ERK2, significant NMR peak intensity changes in residues, such as E81, D82, R85, R89, and G91 (Figs. 3 and 4), together with spin relaxation data (Fig. S1), suggest that this region is highly dynamic—likely influenced by the surrounding flank regions. Docking simulations and AF3 modeling indicate that BCI may initially bind to the surface of MKP3^KBD^, inducing local conformational changes—most notably, an outward shift of the α4-helix (Figs. 4*B* and 7, *A* and *B*). This shift exposes a previously buried hydrophobic pocket, enabling tighter BCI binding. Sequence and structural comparisons further highlight the importance of the α4-helix in BCI selectivity (Figs. 7*C* and S5). MKP4^KBD^, which shares 59% sequence identity with MKP3^KBD^, lacks this helix and instead features a proline-rich region that prevents helix formation, resulting in no detectable BCI binding. In contrast, MKPX^KBD^ and MKP5^KBD^, despite sharing different sequence identities with MKP3^KBD^ (59% and 28%, respectively), retain structural similarity in the α4-helix region and exhibit significant BCI binding. These findings suggest that the α4-helix not only provides a binding interface but also undergoes dynamic conformational changes essential for accommodating BCI. Future experimental validation, including targeted mutagenesis and high-resolution structural studies, will be necessary to fully elucidate the role of the α4-helix in BCI binding across MKPs and to guide the rational design of MKP-targeted inhibitors.

Beyond MKP3, BCI has also been reported to inhibit MKP1 while sparing phosphatases, such as CDC25, PTP1B, and VHR (DUSP3) (29). However, our investigation into BCI’s selectivity across the MKP family reveals that BCI binds significantly to the KBDs of MKP3, MKPX, and MKP5 but exhibits weak or no interaction with the KBDs of MKP1, MKP2, and MKP4, contradicting previous reports (29, 30). The reported MKP1–BCI interaction was based on a cell-based chemical complementation assay without direct *in vitro* validation. Moreover, MKP1 primarily dephosphorylates JNK and p38 MAPKs rather than ERK1/2 (46, 47, 48), suggesting that BCI may inhibit MKP1 and MKP3 through distinct mechanisms.

The high sequence conservation of MKPs’ CD has long hindered the development of selective inhibitors targeting this region, resulting in limited success to date (7, 26, 28). In contrast, targeting the KBD presents a promising alternative for modulating phosphatase activity. This strategy is particularly compelling given the persistent challenges in designing catalytic site–directed MKP inhibitors with high specificity. Unlike conventional catalytic inhibitors, which often suffer from poor selectivity because of conserved active sites, KBD-targeting compounds may enable selective inhibition of phosphatases that rely on MAPK docking interactions. The ability of BCI to disrupt MKP3–ERK2 binding supports this concept and suggests that similar strategies could be applied to other MKPs—such as MKP1, MKPX, and MKP5—that regulate ERK and/or p38 MAPKs through docking-dependent mechanisms (10, 13, 16, 49, 50).

Our findings also carry important implications for drug development. Although BCI exhibits relatively low affinity (∼15 μM), it serves as a proof-of-concept molecule, demonstrating the feasibility of targeting the KBD for selective phosphatase inhibition. The development of higher-affinity analogs could lead to more potent inhibitors with therapeutic potential. Furthermore, this strategy could be extended beyond MKPs to other phosphatases and kinases with regulatory docking domains, broadening its relevance to signaling pathways implicated in cancer, neurodegeneration, and immune regulation. To further support this approach, we successfully predicted the structure of the MKP3^KBD^–BCI complex using AF3, guided by NMR and MST data, as crystallization of the complex was unsuccessful. Remarkably, the predicted model aligned well with the binding sites identified by NMR titration, highlighting AF3 as a reliable and effective tool for modeling protein–ligand interactions in the absence of high-resolution structural data. This integrative strategy underscores the growing potential of combining experimental biophysical techniques with artificial intelligence–based modeling to accelerate structure-based inhibitor design (51, 52, 53).

In conclusion, our study identifies the KBD as a novel and druggable site for MKP3 inhibition, shifting the focus away from traditional catalytic inhibitors toward a new class of allosteric phosphatase modulators. By demonstrating that BCI disrupts MKP3–ERK2 interactions rather than directly inhibiting catalytic activity, we provide a framework for the development of selective MKP inhibitors that may have broader applications in targeting MAPK-driven diseases. This work lays the foundation for future efforts in optimizing KBD-targeting molecules for therapeutic intervention.

## Experimental procedures

### Protein expression and purification

The FL, KBD, or CD of human MKPs, including MKP3(1–381) (MKP3^FL^), MKP3(1–154) (MKP3^KBD^), MKP3(14–152), MKP3(204–350) (MKP3^CD^), MKP1(1–141) (MKP1^KBD^), MKP2(5–159) (MKP2^KBD^), MKPX(1–140) (MKPX^KBD^), MKP4(1–143) (MKP4^KBD^), MKP5(139–287) (MKP5^KBD^), and MKP5(320–467) (MKP5^CD^), were cloned into either pET21b, pET15b, or pPH vectors (Novagen). Specially, pET21b carries a C-terminal His_6_ tag (C-His_6_), pET15b contains an N-terminal His_6_ tag (N-His_6_) followed by a thrombin cleavage site, and pPH, derived from pETDuet-1, includes an N-terminal glutathione-*S*-transferase (GST) tag (N-GST) followed by a PreScission protease cleavage site. Mouse ERK2(9–356) was cloned into the pPH vector. To produce bis-pERK2 phosphorylated at T183 and Y185, ERK2 and its constitutively active upstream kinase MEK1EEΔN4 were coexpressed from the same pETDuet-1-GST plasmid, which is derived from pETDuet-1 (Novagen) and carries an N-terminal GST tag followed by a thrombin cleavage site. Various MKP3^FL^ mutants were generated using a site-directed mutagenesis kit (QuickChange; Agilent). All constructs were verified by DNA sequencing. Plasmid information is summarized in Table S1.

Proteins were expressed in *Escherichia coli* BL21(DE3). Cells were grown in LB medium supplemented with 0.1 mg/ml ampicillin at 37 °C with shaking at 200 rpm until the absorbance reached 0.7 to 1.0 at 600 nm, typically after 2.5 to 3 h. Protein expression was then induced with 0.2 mM IPTG at 16 °C for 16 h. Cells were harvested by centrifugation at 4000 rpm for 10 min at 4 °C and stored at −80 °C until use. Uniformly ^13^C- and/or ^15^N-labeled proteins were produced by initially growing cells in LB medium to an absorbance of ∼0.8 at 600 nm, followed by centrifugation at 5000*g* for 7 min at 4 °C. The cell pellets were then resuspended and transferred to M9 minimal medium at a 2:1 volume ratio (2 l LB culture to 1 l M9 medium), supplemented with ^15^NH_4_Cl (1 g/l) and/or ^13^C-glucose (2.5 g/l) (Cambridge Isotope Laboratories) as the sole nitrogen and carbon sources, respectively.

Cell pellets were lysed by ultrasonication in an ice-water bath using a buffer containing 50 mM Tris–HCl (pH 8.0), 1 mM Tris(2-carboxyethyl)phosphine hydrochloride, and 0.2 to 1 M NaCl, with the NaCl concentration adjusted according to the construct: 0.2 M for MKP3(1–154), MKP3(14–152), MKP3(204–350), MKP1(1–141), MKP2(5–159), MKPX(1–140), and MKP4(1–143); 0.3 M for MKP3(1–381), MKP5(320–467), ERK2, and pERK2; and 1 M for MKP5(139–287). The lysates were clarified by centrifugation at 13,000 rpm and 4 °C for 40 min, and the supernatants were collected for purification. Proteins with GST or His_6_ tags were purified using GST affinity columns (GE Healthcare) or Ni–NTA (Qiagen) affinity columns, respectively. For NMR measurements, proteins were further treated with thrombin or PreScission protease at 4 °C for 2 h to remove the His_6_ tag. For MST experiments, the His_6_ tag was retained. Both cleaved and uncleaved proteins were subsequently purified by size-exclusion chromatography using Superdex 75 or Superdex 200 columns (GE Healthcare). The elution buffer was selected according to the requirements of subsequent experiments. Protein purity was assessed by SDS-PAGE gel stained with Coomassie Brilliant Blue (Fig. S6), and the yields, determined based on an absorbance at 298 nm, are summarized in Table S1. The purified proteins were either used immediately or flash-frozen in liquid nitrogen for storage at −80 °C.

### NMR spectroscopy

NMR data were collected at 298 K on a Bruker AVANCE NEO 800 MHz spectrometer (Bruker BioSpin) equipped with a ^1^H&^19^F/^13^C/^15^N TCI cryogenic probe. Backbone resonances of MKP3^1–154^ were assigned using 2D/3D experiments, including 2D ^1^H–^15^N HSQC and 3D ^1^H/^13^C/^15^N CBCA(CO)NH, HNCACB, HNCO, and HN(CA)CO, performed with uniformly ^13^C/^15^N labeled protein at a final concentration of 0.39 mM in buffer containing 20 mM imidazole (pH 6.0), 100 mM NaCl, 5 mM DTT, 0.5 mM EDTA, and 5% (v/v) D_2_O. The buffer used for backbone resonance assignment was similar to that used for the previously reported NMR structure (15). NMR titration experiments were conducted using ^15^N-labeled MKP3^1–154^, MKP3^14–152^, MKP3^204–350^, MKP1^1–141^, MKP2^5–159^, MKPX^1–140^, MKP4^1–143^_,_ and MKP5^139–287^. 2D ^1^H–^15^N HSQC spectra of ^15^N-labeled MKPs^KBD^ (0.1 mM) were acquired in the absence and presence of BCI (50 mM stock in DMSO-d6) in buffer containing 50 mM KH_2_PO_4_–K_2_HPO_4_ (pH 7.2), 100 mM KCl, 1 mM Tris(2-carboxyethyl)phosphine hydrochloride, 1 mM EDTA, and 5% (v/v) D_2_O. Control experiments, in which only the corresponding volume of DMSO-d6 was added to ^15^N-labeled proteins, were performed to exclude possible effects of DMSO on the interactions. For ^15^N T_1_ measurements, the relaxation delay times were set to 20 (×2), 100, 200 (×2), 300, 400, 600 (×2), 800, 1200, and 1600 ms. For ^15^N T_2_ measurements, the delay times were 7.84, 15.68 (×2), 23.52, 31.36 (×2), 39.2, 47.04 (×2), 54.88, 62.72, 78.4, 94.08, and 109.76 ms. The relaxation rates R_1_ (1/T_1_) and R2 (1/T_2_) for each residue were determined by fitting the peak intensities from spectra acquired at the various delay times to a single-exponential decay function: *I*/*I*_0_ = exp(-R_1,2_·*t*), where *I*_0_ is the intensity at *t* = 0, and *I* is the intensity at a delay time *t*. Repeated measurements were used to estimate the uncertainties of the R_1_ and R_2_ values *via* Monte Carlo analysis. Heteronuclear steady-state ^1^H–^15^N NOE experiments were acquired in an interleaved mode, with 5 s of ^1^H saturation for NOE build-up and 5 s of relaxation delay for the reference spectra. Repeated experiments were performed to estimate uncertainties, and the propagated error for each NOE value was calculated using the following equation: *σ*_NOE_ = NOE × ((*σ*_sat_/*I*_sat_)^2^+(*σ*_unsat_/*I*_unsat_)^2^)^1/2^, where *σ*_sat_ and *σ*_unsat_ are the SDs of the saturated and unsaturated spectra, respectively, and *I*_sat_ and *I*_unsat_ are the corresponding signal intensities. NMR data were processed using TopSpin 4.1.1 (Bruker BioSpin) or NMRPipe (54) and analyzed with POKY (55).

### MST affinity measurements

MST measurements were performed using fluorescently labeled proteins and the inhibitor BCI (CAS: 1245792-51-1; TargetMol) on a Monolith instrument (NanoTemper Technologies). The RED–Tris–NTA second-generation dye was dissolved in MST buffer (20 mM Hepes, pH 7.4, 200 mM NaCl, and 0.05% Tween-20) to prepare a 5 μM stock solution. Fluorescent labeling of MKP proteins followed the standard protocol of the MO-L018 RED–Tris–NTA protein labeling kit, leveraging the His-tag–dye interaction.

The BCI compound was prepared as a 600 to 750 μM stock solution in MST buffer with 10% DMSO to enhance solubility, followed by a 16-point serial dilution. The diluted BCI solutions were thoroughly mixed with labeled MKP proteins (50 nM) at a 1:1 volume ratio, yielding a final volume of 10 μl. The reaction mixtures were incubated in the dark for 10 min at room temperature before being loaded into standard glass capillaries. Measurements were carried out on the Monolith instrument (100% LED, 40% IR laser power) with a 20 s laser on time. The normalized fluorescence values from triplicate measurements were averaged at each concentration and fitted to a 1:1 binding model using the KD model in MO.Affinity software (v3.0.5, NanoTemper Technologies) to determine the binding affinities between MKP proteins and the BCI inhibitor. The final reported *K*_*d*_ value represents the mean ± SD of *K*_*d*_ values obtained from three independent experiments, each fitted separately.

### Phosphatase activity assays

The phosphatase activity of MKP3 toward pNPP was measured using continuous spectrophotometric assays, either in the absence or the presence of ERK2 at a 1:1 M ratio with MKP3. Experiments were conducted at 25 °C in a reaction mixture with a total volume of 1.8 ml, containing 50 mM Mops (pH 7.0), 100 mM NaCl, and 0.1 mM EDTA. MKP3 (1 μM) was preincubated with varying concentrations of the inhibitor BCI for 5 min before initiating the reaction by adding pNPP at concentrations ranging from 0 to 14 mM. Continuous absorbance changes were recorded using a PerkinElmer LAMBDA 45 spectrophotometer equipped with a magnetic stirrer in the cuvette holder. The production of *p-*nitrophenol was monitored at 410 nm using an extinction coefficient of 18,000 M^-1^ cm^-1^.

The phosphatase activity of MKP3 (0.5 μM) toward pERK2 (0.5 μM) was measured using a coupled assay, in which phosphate released from the substrate was transferred to 7-methyl-6-thioguanosine (MESG) by purine nucleoside phosphorylase. Assays were performed in a reaction mixture containing 50 mM Mops (pH 7.0), 100 mM NaCl, 0.1 mM EDTA, 100 μM MESG, 4 μM purine nucleoside phosphorylase, and varying concentrations of BCI, as indicated in Figure 5. Dephosphorylation was monitored by measuring absorbance changes at 360 nm, reflecting the conversion of MESG to 7-methyl-6-thioguanine in the presence of inorganic phosphate. Phosphate quantification was performed using an extinction coefficient of 11,200 M^-1^ cm^-1^ at 360 nm.

Stock solutions of BCI (dissolved in DMSO) at different concentrations were prepared to maintain a constant DMSO concentration across all BCI concentrations in the phosphatase inhibition assays, thereby eliminating potential effects of varying DMSO levels on detection. Initial reaction rates were determined from the linear slope of progress curves, and the data were analyzed using nonlinear regression. Kinetic parameters (*k*_cat_ and *K*_*m*_) were obtained by fitting the data to the Michaelis–Menten equation: *v*_0_ = *k*_cat_[E][S]/(*K*_*m*_ + [S]). Each *v*_0_ value, corresponding to a specific substrate concentration, represents the mean of three independent measurements, with errors reported as SD. These mean *v*_0_ values were used for nonlinear fitting, and the errors in *k*_cat_ and *K*_*m*_ were derived from the SEs of the fitted parameters.

### Docking simulations

Virtual simulations were performed using Discovery Studio (v3.5; Accelrys) with the AF2-predicted structure of MKP3^KBD^ and the BCI compound structure obtained from PubChem (CID: 6419844). Molecular docking of BCI with MKP3^KBD^ was carried out using CDOCKER, a CHARMM-based molecular dynamics docking algorithm. Briefly, the protein structure was prepared using the “Prepare Protein” module, and the binding site was identified using the ”From Receptor Cavities” option with a site radius of 10 Å. The ligand was preprocessed sequentially using the “Minimized Ligand” and "Prepare Ligand” modules before docking. The prepared ligand was then docked into the receptor using the default CDOCKER parameters.

### AF predictions

The apo structures of MKPs^KBD^ and the structure of the MKP3^KBD^–ERK2 complex were predicted using AF2. BCI-bound MKPs^KBD^ structures were modeled using AF3 with the BCI structure obtained from PubChem (CID: 6419844). The interface predicted template modeling and ranking scores for the AF3 models were as follows: 0.84 and 0.85 for MKP1^KBD^–BCI, 0.80 and 0.81 for MKP2^KBD^–BCI, 0.68 and 0.73 for MKP3^KBD^–BCI, 0.83 and 0.83 for MKP5^KBD^–BCI, and 0.76 and 0.77 for MKPX^KBD^–BCI models. All structures were visualized using the PyMOL Molecular Graphics System (v3.0; Schrödinger).

## Data availability

All data are contained within the article.

## Supporting information

This article contains supporting information.

## Conflict of interest

The authors declare that they have no conflicts of interest with the contents of this article.
