# Supplementary material for: BCI inhibits MKP3 by targeting the kinase-binding domain and disrupting ERK2 interaction
Source: J Biol Chem. 2025 Aug 6;301(9):110570. doi: 10.1016/j.jbc.2025.110570 (PMC12444472; doi:10.1016/j.jbc.2025.110570)
Supplement: Supplementary Material [file mmc1.docx]

**BCI inhibits MKP3 by targeting the kinase-binding domain and disrupting ERK2 interaction**

**Su-Jie Qiu^1,2^, Ya-Liang Zhang^3^, Wei-Bin Gong^4^, Yu-Han Ding^1^, Jia-Wei Wu^1,2^, Zhi-Xin Wang^1,2*^, Hong-Wei Yao^1,2*^**

^1^Institute of Molecular Enzymology, School of Life Sciences, Suzhou Medical College of Soochow University, Suzhou, Jiangsu, P.R. China

^2^MOE Key Laboratory of Geriatric Diseases and Immunology, Suzhou Medical College of Soochow University, Suzhou, Jiangsu, P.R. China

^3^State Key Laboratory of Pharmaceutical Biotechnology, School of life Sciences, Nanjing University, Nanjing, Jiangsu, P.R. China

^4^Institute of Biophysics, Chinese Academy of Sciences, Beijing, P.R. China

*Corresponding authors:

Zhi-Xin Wang, Institute of Molecular Enzymology, School of Life Sciences, Suzhou Medical College of Soochow University, Suzhou 215123, P.R. China, Tel: +86-512-65883774, E-mail: zhixinwang@mail.tsinghua.edu.cn

Hong-Wei Yao, Institute of Molecular Enzymology, School of Life Sciences, Suzhou Medical College of Soochow University, Suzhou 215123, P.R. China, Tel: +86-512-65883508, E-mail: hwyao@suda.edu.cn

**Table S1. Protein expression plasmids and yields**

| Protein | Expression plasmid | Tag | Protease cleavage site | Yield (mg per liter of culture) |
| --- | --- | --- | --- | --- |
| MKP3(1-381) | pET21b | C-His_6_ | No | 7.8 |
| MKP3(1-154) | pET15b | N-His_6_ | Thrombin | 9.8 |
| MKP3(14-152) | pET15b | N-His_6_ | Thrombin | 13.2 |
| MKP3(204-350) | pPH | N-His_6_ | PreScission | 11.6 |
| MKP1(1-141) | pPH | N-His_6_ | PreScission | 9.7 |
| MKP2(5-159) | pET21b | C-His_6_ | No | 7.6 |
| MKPX(1-140) | pPH | N-His_6_ | PreScission | 39.6 |
| MKP4(1-143) | pPH | N-His_6_ | PreScission | 5.6 |
| MKP5(139-287) | pET21b | C-His_6_ | No | 10.8 |
| MKP5(320-467) | pPH | N-His_6_ | PreScission | 13.6 |
| ERK2(9-356) | pPH | N-His_6_ | PreScission | 13.1 |
| pERK2(9-356) | pETDuet-1-GST | N-GST | Thrombin | 2.0 |


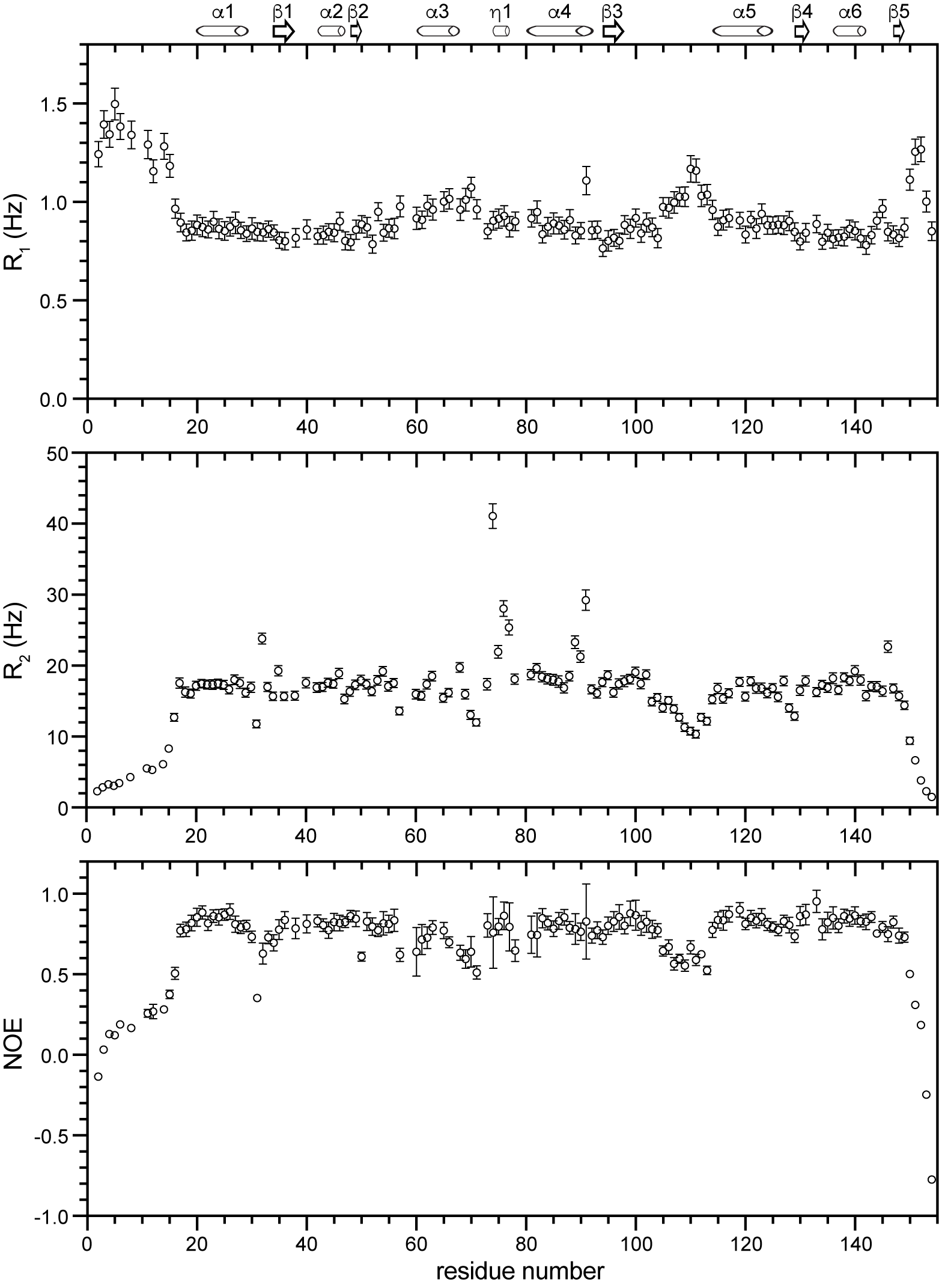


**Figure S1. ^1^H-^15^N relaxation parameters for MKP3(1-154).** Longitudinal relaxation rates (R_1_), transverse relaxation rates (R_2_), and steady-state NOE values are shown for each residue. Secondary structure elements derived from the AlphaFold2 (AF2) model of MKP3(1–154) are indicated at the top.


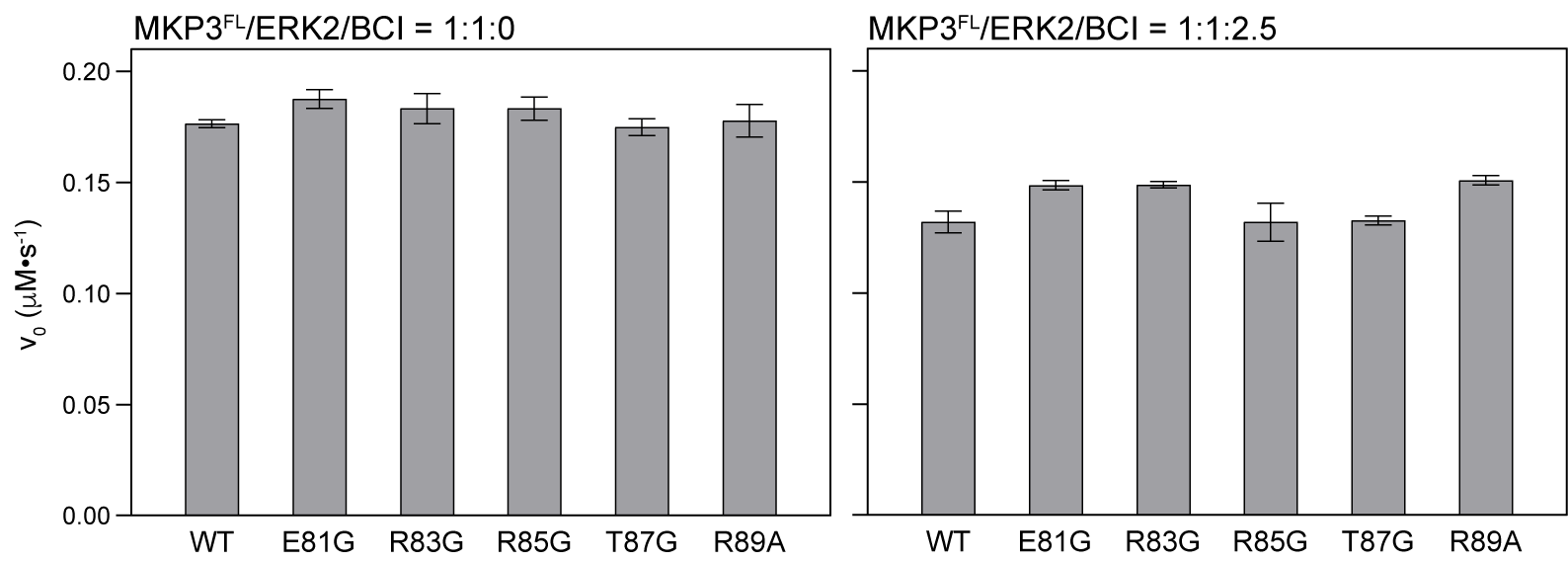


**Figure S2. Inhibition of full-length MKP3 mutant activity by BCI.** The phosphatase activities (v_0_) of full-length MKP3 and its mutants (E81G, R83G, R85G, R87G, and R89A) at 1 μM concentration were measured using continuous spectrophotometric assays with 8 mM pNPP as the substrate, in the presence of ERK2 (1 μM), either without or with 2.5 μM BCI. Data are presented as mean ± SD (n = 3). Reactions were performed at 25 °C in a total volume of 1.8 mL, in a buffer containing 50 mM MOPS (pH 7.0), 100 mM NaCl, and 0.1 mM EDTA.


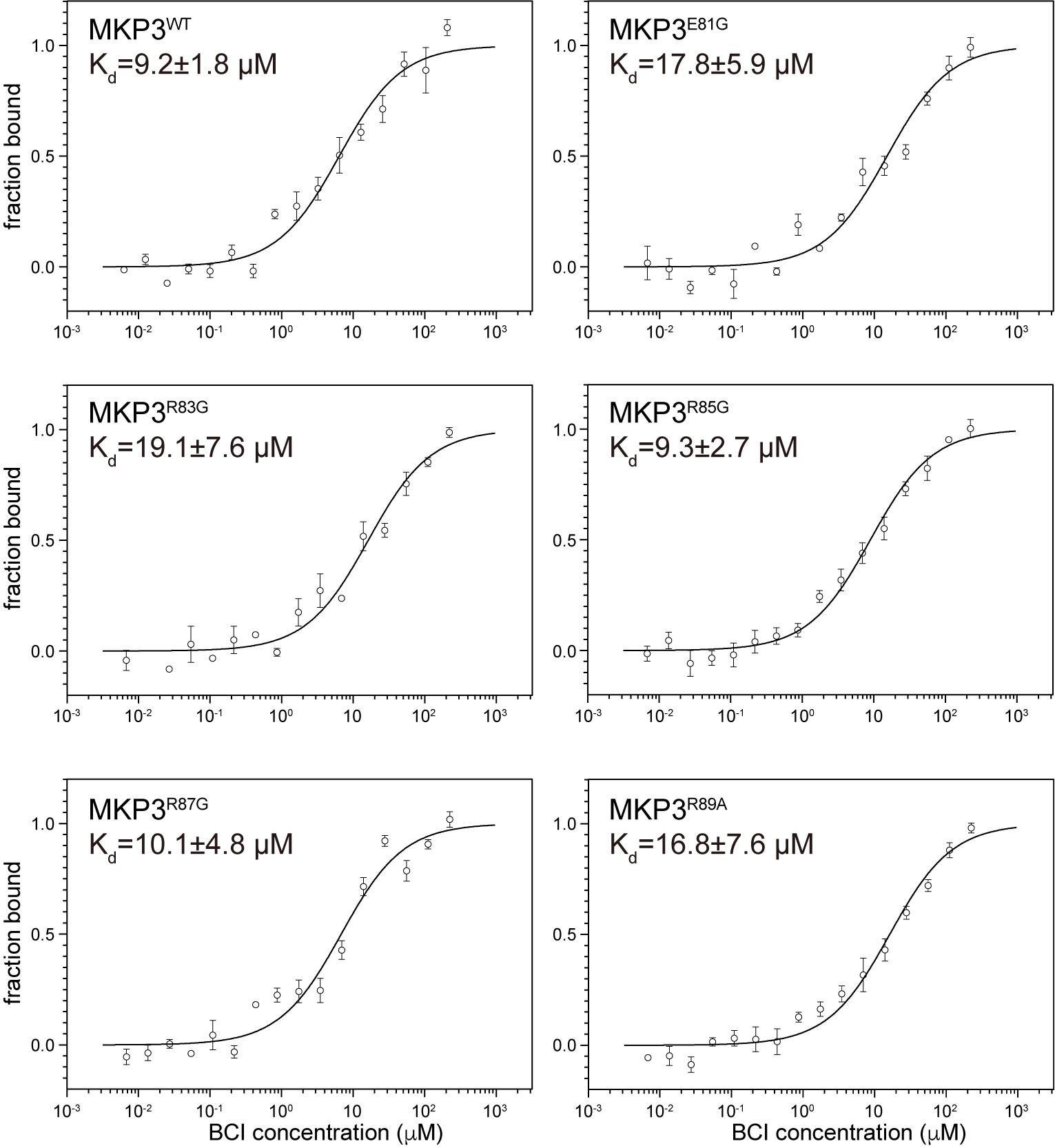


**Figure S3. Affinity measurements of MKP3 mutants with BCI by MST.** The binding affinities of full-length MKP3 mutants (E81G, R83G, R85G, R87G, and R89A) to BCI were determined by MST and are presented as mean ± SD (n = 3). Wild-type MKP3 (MKP3^WT^) was included as a reference.


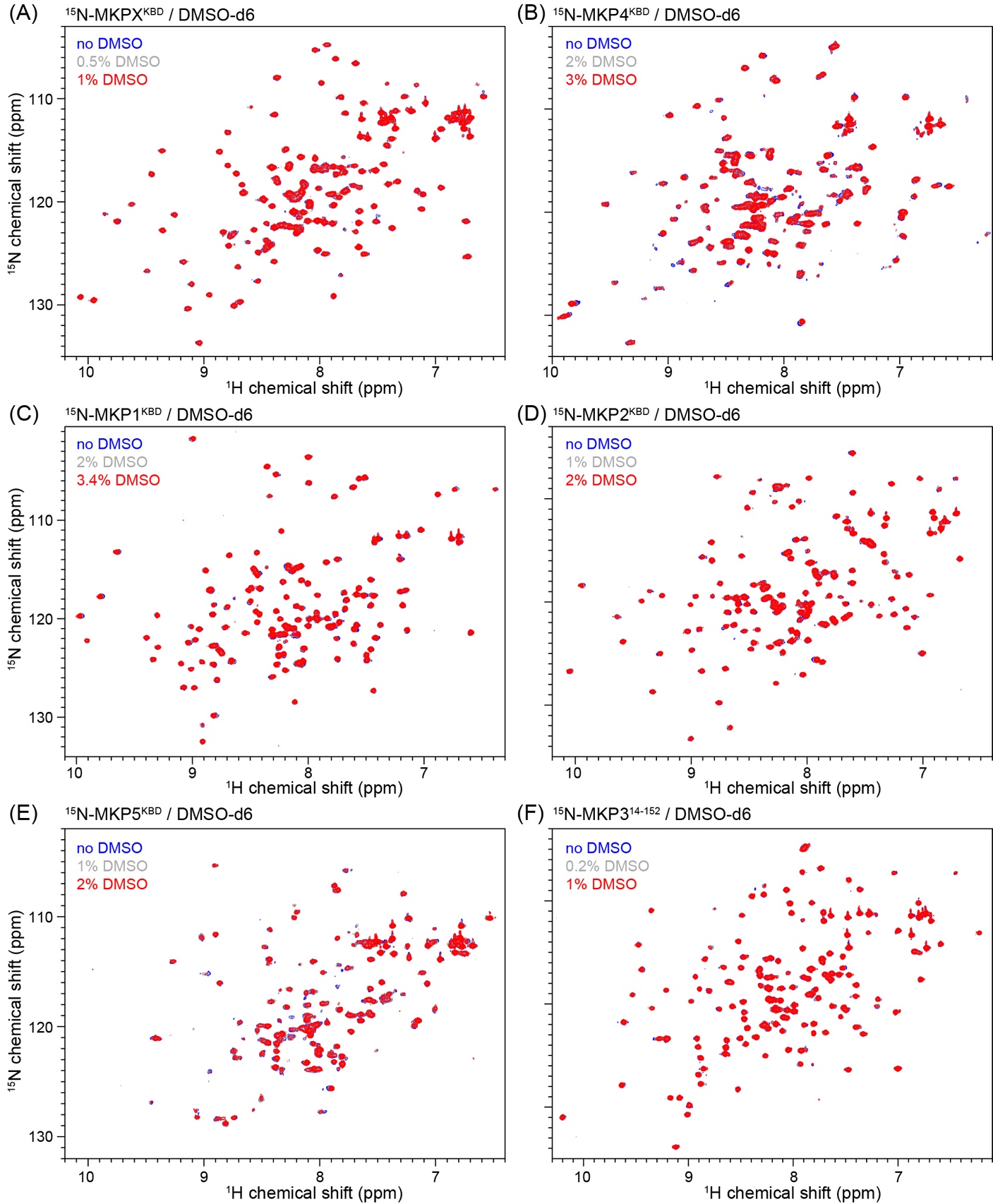


**Figure S4. NMR titrations of MKP phosphatases with DMSO.** (A-F) 2D ^1^H-^15^N HSQC spectra of the kinase-binding domains of various MKP phosphatases in the absence (blue) and presence (gray and red) of deuterated DMSO. The addition of low concentrations of DMSO did not perturb any of the measured NMR HSQC spectra.


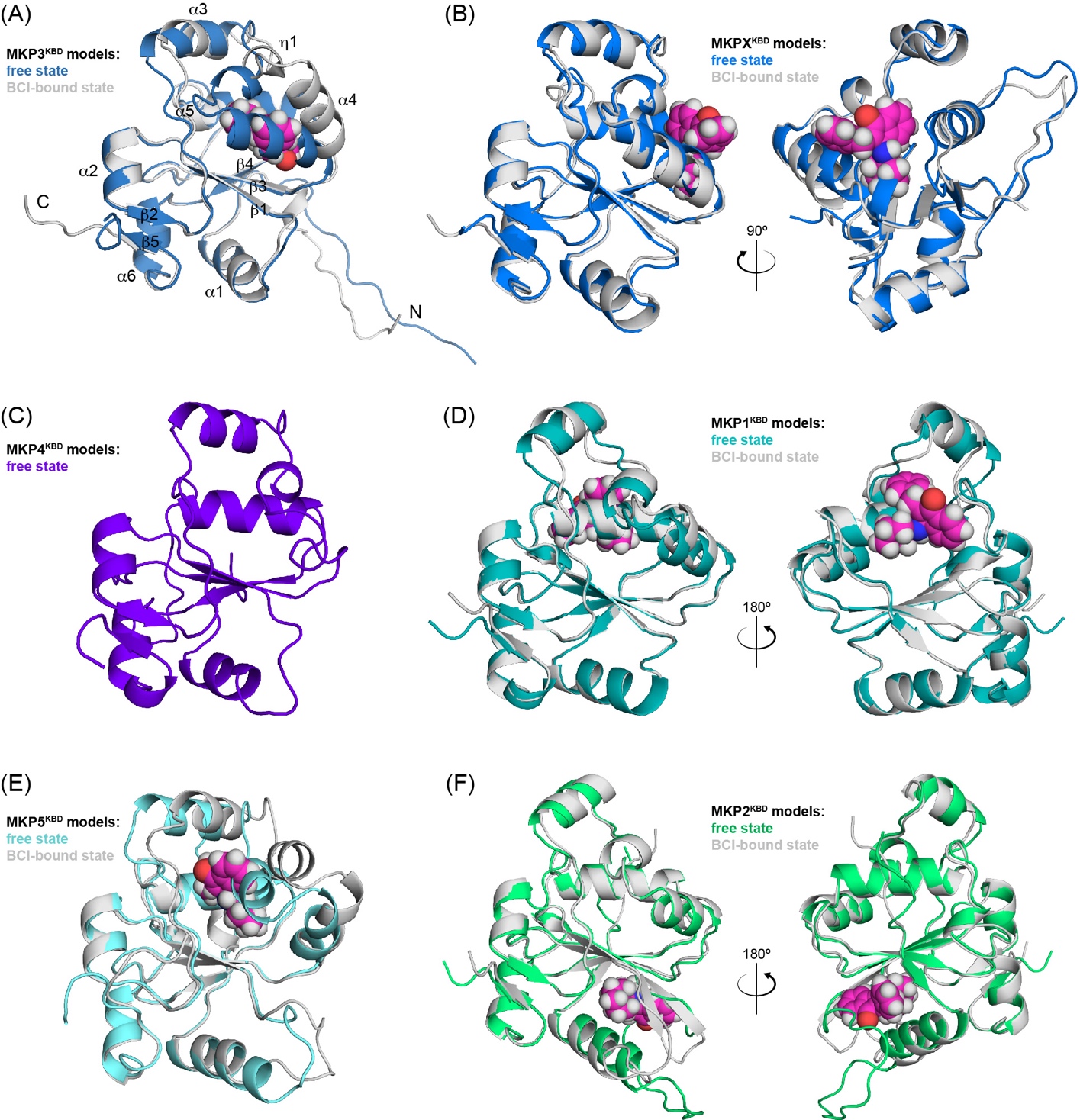


**Figure S5. AF3 models of various MKP^KBDs^ in complex with BCI.** (A-F) Superimposition of structural models in the free and/or BCI-bound states for MKP3^KBD^, MKPX^KBD^, MKP4^KBD^, MKP1^KBD^, MKP5^KBD^, and MKP2^KBD^, respectively. BCI is depicted as spheres with hydrogen, carbon, nitrogen, and oxygen atoms colored gray, magenta, blue, and red, respectively. MKP5^KBD^ exhibits BCI-binding pocket similar to that of MKP3^KBD^, whereas MKPX^KBD^, MKP1^KBD^, and MKP2^KBD^ display distinct BCI-binding pockets.


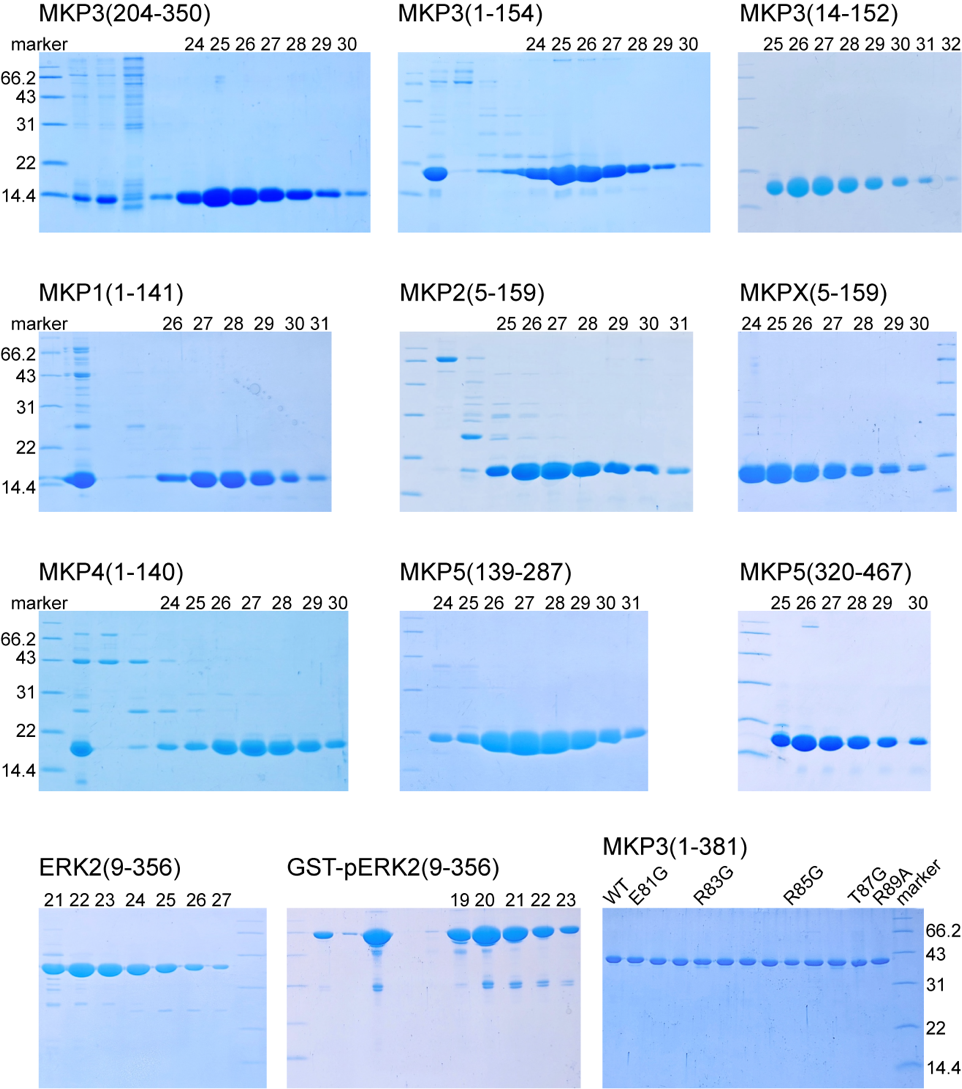


**Figure S6. 15% SDS-PAGE analysis of purified proteins.** Protein purity after size-exclusion chromatography was assessed by 15% SDS–PAGE. Tube numbers corresponding to the collected fractions are labeled above the gel. Wild-type (WT) and single-point mutants of full-length MKP3 are indicated above the gel. Molecular weight markers (in kDa) are shown on the left or right.
